# Rapid, widespread transduction of the murine myocardium using self-complementary Adeno-associated virus

**DOI:** 10.1186/1479-0556-5-13

**Published:** 2007-12-10

**Authors:** Lourdes M Andino, Thomas J Conlon, Stacy L Porvasnik, Sanford L Boye, William W Hauswirth, Alfred S Lewin

**Affiliations:** 1 Department of Molecular Genetics and Microbiology, University of Florida, Gainesville, FL, USA; 2 Powell Gene Therapy Center University of Florida, Gainesville, FL, USA; 3 Department of Pediatrics University of Florida, Gainesville, FL, USA; 4 Department of Ophthalmology, University of Florida, Gainesville, FL, USA

## Abstract

Adeno-associated virus (AAV) has shown great promise as a gene transfer vector. However, the incubation time needed to attain significant levels of gene expression is often too long for some clinical applications. Self-complementary AAV (scAAV) enters the cell as double stranded DNA, eliminating the step of second-strand synthesis, proven to be the rate-limiting step for gene expression of single-stranded AAV (ssAAV). The aim of this study was to compare the efficiency of these two types of AAV vectors in the murine myocardium. Four day old CD-1 mice were injected with either of the two AAV constructs, both expressing GFP and packaged into the AAV1 capsid. The animals were held for 4, 6, 11 or 21 days, after which they were euthanized and their hearts were excised. Serial sections of the myocardial tissue were used for real-time PCR quantification of AAV genome copies and for confocal microscopy. Although we observed similar numbers of AAV genomes at each of the different time points present in both the scAAV and the ssAAV infected hearts, microscopic analysis showed expression of GFP as early as 4 days in animals injected with the scAAV, while little or no expression was observed with the ssAAV constructs until day 11. AAV transduction of murine myocardium is therefore significantly enhanced using scAAV constructs.

## Results and discussion

Adeno-associated virus has become an important tool for gene transfer because of its lack of pathogenicity and its ability to express passenger genes for long periods of time. Although potentially safe as a gene therapy vector, this virus exhibits an extended lag period before transgene expression actually occurs. The reason for this delay in expression is the binding of a cellular protein, FKBP52, to the D-sequence within the inverted terminal repeats (ITRs)[1]. Phosphorylated FKBP52 inhibits viral second strand DNA synthesis, needed for transgene expression, consequently leading to delayed transgene expression [1-3]. As an avenue for bypassing this phenomenon, McCarty *et al*. have introduced the use of a double-stranded form of AAV[4].

The typical single-stranded AAV (ssAAV) genome is flanked by two, 145 bp ITRs. The 3' ITR serves as the replication origin for the viral genome as well as a packaging signal[5]. During replication, AAV genome dimers are formed as replication intermediates. These dimers are subsequently cleaved by AAV Rep proteins at the junction of the ITR and the D sequence. Wang *et al*. discovered that if one of the ITRs has a deleted D-sequence and terminal resolution site (trs), cleavage by Rep cannot occur and consequently, the dimers are not resolved into monomers. Therefore, the double-stranded genomes are then packaged as large hairpin DNA molecules[6].

Although the biology of these constructs has been studied, testing of these constructs for gene delivery to animals is in its early stages. In this report, we demonstrate the efficacy of self complementary AAV (scAAV) in the murine myocardium, a traditionally challenging organ to transduce. Although different viruses have been employed for myocardial gene transfer, each has its own limitations. For example, adenovirus has been shown to be toxic and has short-term expression[7], and lentiviral vectors stimulate inflammatory responses[8]. Although adeno-associated virus has a delay in onset and small packaging capacity, it has been shown to direct gene expression for long periods of time in the heart without any toxicity [9-11].

We compared the expression profile of both the ssAAV and scAAV constructs in murine myocardia using sub-xiphoid injections in 4 day old CD-1 mice (Charles Rivers). The hearts were assessed using direct immunofluorescence of the tissue 4, 6, 11, and 21 days post-injection.

**Figure 1:**
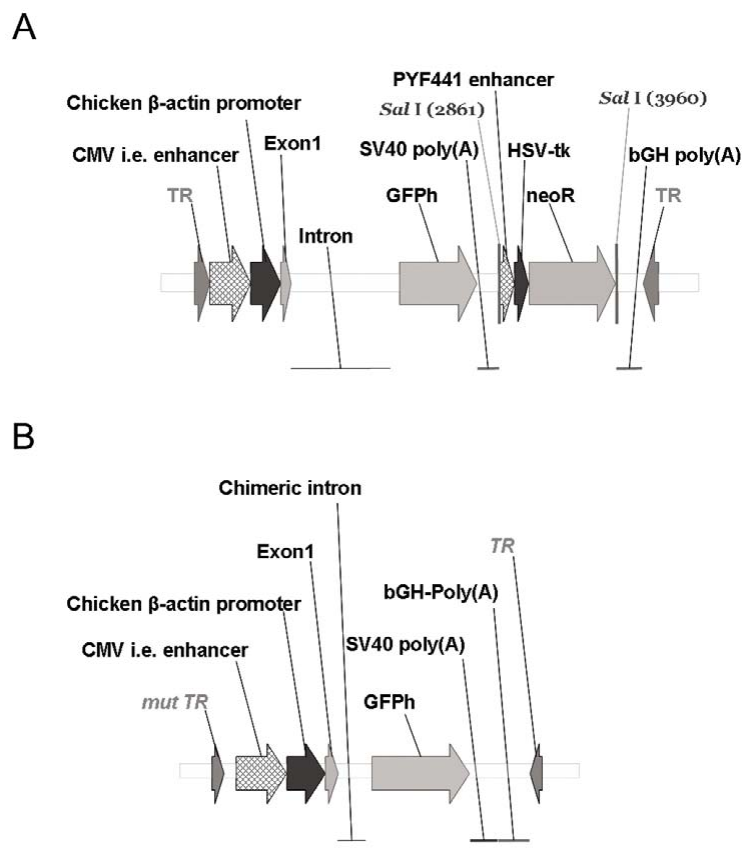
**Maps of scAAV and ssAAV**. (A) A schematic representation of the single-stranded (ss) AAV genome containing a full-length actin intron as well as a Neomycin resistance cassette. This construct contains the CBA promoter driving the expression of GFP. (B) A schematic representation of the self-complementary (sc) AAV genome containing a mutated 5' inverted terminal repeat (ITR), an actin intron from which 722 nucleotides have been deleted, and no Neomycin resistance cassette. This construct also contains the CBA promoter driving the expression of GFP. Both of these constructs contain the AAV2 ITRs and were packaged into AAV1 capsids.

The single stranded GFP-expressing AAV vector (ssAAV) contained 4.3 kb of DNA surrounded by AAV-2 inverted terminal repeats (ITR) (Figure 1A). Expression of GFP was driven by the CMV enhancer-chicken β-actin promoter (CBA) and contained the β-actin exon and the corresponding 924 bp intron. Downstream of this was the humanized GFP (GFPh) gene followed by a 1099 bp Neomycin resistance cassette flanked with *Sal*I cut sites. The second plasmid vector used was very similar to ssAAV in that it also expressed the GFPh gene under the control of the CBA promoter (Figure 1B). This vector lacked the Neomycin resistance cassette. Instead of the full length intron and exon this construct had an intron of 202 bp containing the donor and acceptor sites necessary for splicing. Additionally, as described by Wang *et al*., the 5' ITR had the D-sequence and the trs site removed to prevent cleavage, and resolution of dimers by AAV Rep[6]. Therefore, the total region to be packaged was 2,438 bp, making it suitable to be packaged as a double-stranded or self-complementary molecule (scAAV). The DNA from both vectors was packaged according to previously reported methods[12] into AAV1 capsids which are known to efficiently transduce myocardial tissue[13].

Once the constructs were packaged, four day old CD-1 mice (Charles Rivers) were anesthetized on ice until movement was barely visible, and injections into the cardiac chamber were carried out as described by Zhang *et al*[14]. Each animal was injected with 1.85 × 10^11 ^total particles of AAV1 containing either the ssAAV or the scAAV expression cassettes. Three animals were injected per group. Animals were returned to their dams for 4, 6, 11, or 21 days and then euthanized. Their hearts were extracted and then cut into thirds representing the apical region, the middle region and the base region of the heart. The tissue was then fixed for 12 hours with 4% paraformaldehyde, rinsed in PBS and then allowed to equilibrate in 20% sucrose for 12–24 hours. The next day, hearts were frozen in OCT compound (Tissue-Tek). Five micron sections were cut in a cryostat and used for direct visualization of GFP. Slides were then mounted using Vectashield mounting media containing DAPI (Vector Labs). Two sections, each containing 75 μm of tissue, were reserved for quantitative real-time PCR. Because of its high content of adenine dinucleotides[15], cardiac tissue exhibits a high level of autofluorescence and this background can obscure the detection of GFP even when secondary antibodies are employed[16]. Therefore, we chose to measure the direct GFP signal generated using confocal microscopy of the fixed tissues. This technique allowed us to almost entirely eliminate background fluorescence seen with conventional fluorescent microscopes and filters enabling us to accurately represent the transduced cells. A Leica confocal microscope was used to obtain fluorescent images. Montages consisting of 2–4 fields of view per section were created using Adobe Illustrator CS2 and Adobe Photoshop CS2.

**Figure 2:**
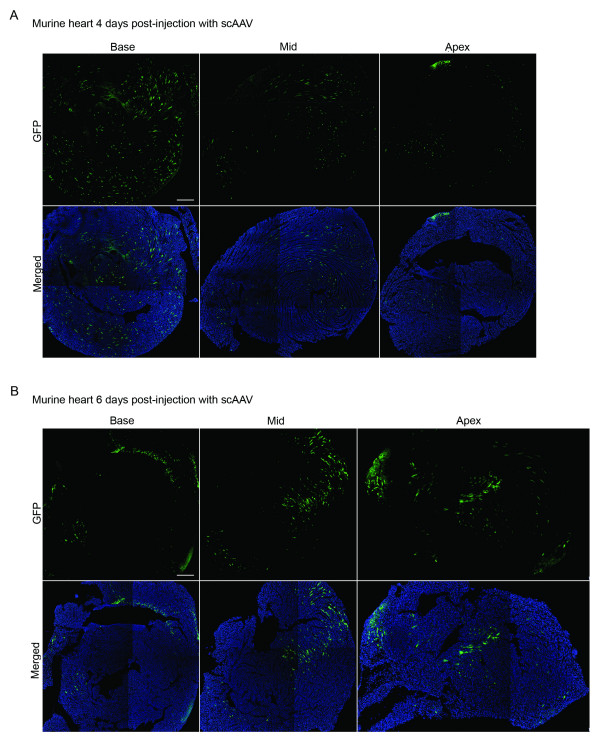
**Rapid onset of gene expression using scAAV in the murine heart**. CD-1 mice (Charles Rivers) were injected 4 days after birth with 1.85 × 10^11 ^vector genomes (vg) of scAAV using previously established methods [14]. Animals were returned to their dams for 4 days (A) or 6 days (B) at which time their hearts were harvested, cut into thirds representing the apical region (apex), the mid-region (mid), or base of the heart (base). The tissues were fixed in 4% paraformaldehyde for 12 hours, rinsed in PBS and allowed to equilibrate in 20% sucrose for 12–24 hours. The next day, the hearts were frozen in cryomolds containing OCT compound (Tissue-Tek) to prepare for cryostat sectioning into 5 μm sections. Slides were mounted using Vectashield mounting media containing DAPI (Vector Labs) to counter stain the nuclei. A Leica TCS SP2 AOBS Spectral Confocal Microscope with a 10× objective was used to obtain fluorescent images. Staining was documented using the Leica Confocal Software (LCS) Version 2.61. Sections with GFP expression can be seen in the top panels while merged images containing GFP and DAPI signals can be seen in the lower panels. The bar represents 150 μm in the 6 day apex and 300 μm in all other panels.

**Figure 3:**
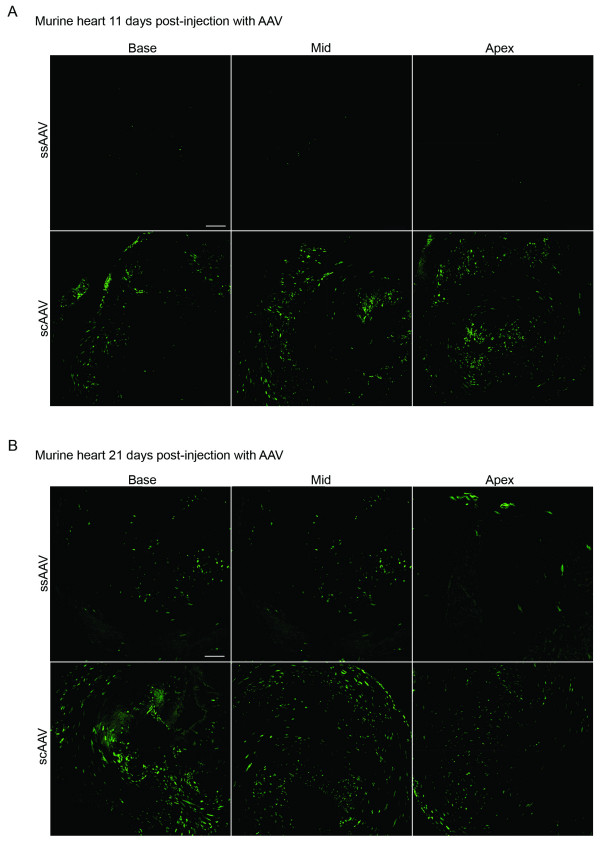
**Increased gene expression with scAAV after longer intervals**. CD-1 mice (Charles Rivers) injected 4 days after birth with 1.85 × 10^11 ^vector genomes (vg) of either scAAV or ssAAV using sub-xiphoid injections [14]. The animals were returned to their dams for 11 days (A) or 21 days (B) and then processed as described in Figure 2. Representative sections with GFP fluorescence are pictured. The bar represents 150 μm in the 21 day ssAAV-apex and 300 μm in all other panels.

Four days post-injection, scAAV treated animals showed widespread GFP expression throughout the myocardium (Figure 2A) while ssAAV injected animals failed to reveal any GFP expression (data not shown). Similarly, 6 days post-injection GFP expression was detected throughout the myocardium in scAAV injected mice (Figure 2B) while the ssAAV injected animals exhibited no GFP expression (data not shown). On the 11^th ^day post-injection, a few GFP positive cells were visible in the ssAAV treated hearts (Figure 3A, top panels), while very high levels of GFP were clearly visible in the apical, mid-section, and regions of the base of scAAV treated hearts (Figure 3A, bottom panels). At 21 days post-injection, GFP expressing cells were apparent in the ssAAV infected hearts throughout the myocardium (Figure 3B, top panels) although at a much lower level than scAAV treated hearts (Figure 3B, bottom panels) which had GFP expression throughout all three regions of the myocardium.

**Figure 4:**
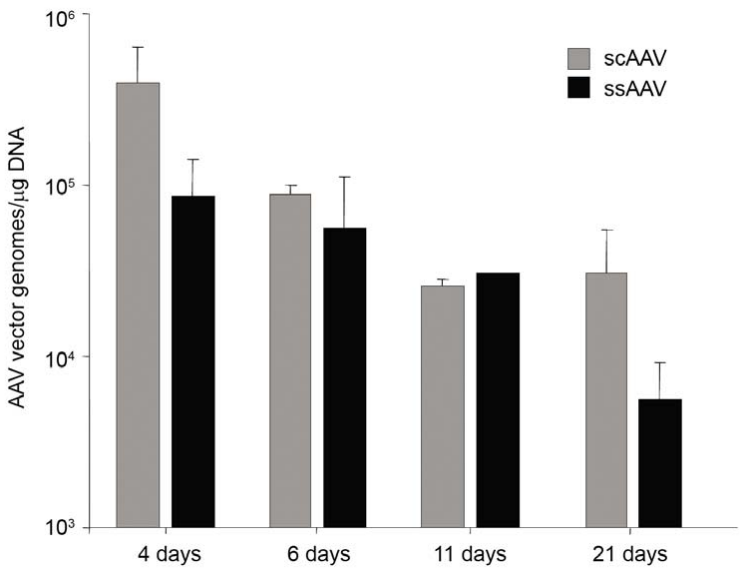
**Similar copy numbers of ssAAV and scAAV in infected hearts**. Serial sections of tissues used for microscopy were collected for real-time PCR analysis of AAV vector genomes. Genomic DNA (gDNA) was extracted from the tissues using a Qiagen DNeasy tissue kit according to the manufacturer's protocol. One microgram of extracted gDNA was used in all quantitative PCR reactions. The PCR conditions were 50 cycles of 94.8°C for 40 s, 37.8°C for 2 min, 55.8°C for 4 min, and 68.8°C for 30 sec. DNA samples were assayed in triplicate. The third replicate was spiked with CBA DNA at a ratio of 100 copies/μg of gDNA. If at least 40 copies of the spike-in DNA were detected, the DNA sample was considered acceptable for reporting vector DNA copies. Data were averaged by group and plotted with standard deviation for comparisons. No statistically significant differences were measured between any of the groups of scAAV and ssAAV treated animals.

One explanation for the increased expression of GFP using scAAV is increased viral infection using that preparation relative to the ssAAV preparation. To determine genome copy number in infected hearts, genomic DNA (gDNA) was extracted from serial sections adjacent to the ones used for immunofluorescence using the Qiagen DNeasy tissue kit. One microgram of extracted gDNA was used in all quantitative polymerase chain reactions. Primer pairs were designed to the CBA promoter and standard curves established by spike-in concentrations of a plasmid DNA containing the same promoter. DNA samples were assayed in triplicate. The third replicate was spiked with CBA DNA at a ratio of 100 copies/μg of gDNA. If at least 40 copies of the spike-in DNA were detected, the DNA sample was considered acceptable for reporting vector DNA copies. Results from these experiments indicated that although there were differences in the amount of GFP directly visualized using immunofluorescence, the amount of AAV genome copies found in the scAAV or ssAAV infected hearts were similar (Figure 4). There was no statistical significance between the amounts of vector genomes found in the scAAV infected heart versus an ssAAV infected heart. A gradual decline in AAV vector genomes observed over the time course of the experiment could be attributed to the dilution of the viral genomes as the heart grew within the developing pups as well as due to natural cell death that occurs during development.

We have described a novel self-complementary AAV vector that contains the chicken β-actin promoter driving the expression of GFP. Despite similar number of vector genomes observed in hearts infected with either scAAV or ssAAV, the scAAV transduced animals showed robust and widespread GFP expression as early as 4 days post-injection, while even at 11 days, expression using ssAAV was minimal. GFP expression could be seen all throughout the heart from the base to the apex. The amount of GFP expression increased over the time course of the experiment in both of the scAAV and ssAAV transduced animals. Nonetheless, the amount of GFP expression seen in the scAAV injected animals was much higher than the ssAAV injected animals at each time point.

Although this construct will not be suitable for packaging large transgenes, this type of vector will be very useful for the delivery of small transgenes and small molecules such as ribozymes and shRNA molecules. Additionally, Wu *et al*. have demonstrated that the packaging capacity of these self-complementary vectors can be as large as 3.3 kb which is more than what was originally expected[17]. If efficient myocardial transduction can be demonstrated in adult animals, these double-stranded AAV vectors might ultimately prove useful in patients who need rapid expression of therapeutic genes.

## Competing interests

We would like to disclose that William W. Hauswirth, contributing author, and the University of Florida own stock in the AGTC Corporation, which develops AAV technology.

## Authors' contributions

LMA was involved in the conceptual design and acquisition of data. TJC assisted by performing quantitative real-time PCR of vector genomes in cardiac tissue. SLP was involved in tissue harvesting and processing. SLB generated the novel scAAV construct with the short chicken B-actin promoter. WWH and ASL were the coordinators of the project.
